# Uncommon pair: tonsillar carcinoma and Abrikossoff tumor—case report and review

**DOI:** 10.3389/fonc.2025.1560133

**Published:** 2025-06-10

**Authors:** Filippo Valentini, Daniela Messineo, Mara Riminucci, Alessandro Corsi, Armando De Virgilio

**Affiliations:** ^1^ Department of Sense Organs, Sapienza University of Rome, Rome, Italy; ^2^ Department of Radiologic, Oncologic and Anatomo-Pathologic Sciences, Sapienza University of Rome, Rome, Italy; ^3^ Department of Molecular Medicine, Section of Pathology, Sapienza University of Rome, Rome, Italy

**Keywords:** tonsillar carcinoma, lymphoepithelial carcinoma (LEC), transoral robotic surgery (TORS), Abrikossoff tumor, vocal cord abnormalities, head and neck squamous cell carcinoma (HNSCC), oropharyngeal cancer (OPSCC), tumor board

## Abstract

Lymphoepithelial carcinoma (LEC) is a rare malignancy within the head and neck region, histologically akin to undifferentiated nasopharyngeal carcinoma. It is uncommon outside the nasopharynx, particularly in the oropharynx and salivary glands. Granular cell tumors (GCTs), or Abrikossoff’s tumors, are rare mesenchymal neoplasms of neural origin, frequently benign but with the potential for malignant transformation. This case report presents an unprecedented clinical scenario of a 62-year-old female patient diagnosed with an HPV/EBV-negative LEC of the right palatine tonsil, coupled with metachronous squamous dysplasia and a contralateral GCT of the true vocal cord. Following initial presentation with cervical swelling, hoarseness, and dysphagia, diagnostic imaging and histopathological analyses confirmed the distinct co-occurrence of these rare entities. The patient underwent multidisciplinary treatment, including transoral robotic surgery (TORS), radical neck dissection, and adjuvant chemoradiotherapy for LEC. Simultaneously, the GCT was managed surgically with close-margin excision, with ongoing surveillance for recurrence. A comprehensive treatment strategy meticulously addressed the unique pathological and therapeutic challenges posed by the simultaneous management of these neoplasms. This case underscores the critical importance of thorough diagnostic evaluation and multidisciplinary planning in managing complex oncological scenarios. The concurrent presentation of LEC and GCT in the head and neck region necessitates individualized approaches, integrating surgical precision and adjuvant therapies. The rarity of this co-occurrence highlights the need for further studies to refine diagnostic markers and therapeutic guidelines, improving patient outcomes.

## Introduction

Lymphoepithelial carcinoma (LEC) of the head and neck region is a rare malignancy, histologically similar to an undifferentiated non-keratinizing subtype of nasopharyngeal carcinoma (NPC), thus defined by the World Health Organization (WHO) (1), that can be distinguished from a nasopharyngeal lymphoepithelioma by the different ratio in epithelial and lymphoid components, being more precisely described as a lymphoepithelioma-like carcinoma, arising elsewhere other than the nasopharynx. Indeed, this histological subtype of squamous cell carcinoma (SCC) is more stereotyped and well-established in the nasopharyngeal subsite, while its origin elsewhere in the head and neck region is relatively uncommon, with a few cases reported in the literature of primary occurrence in the oropharynx, oral cavity, and salivary glands (palatine tonsils and tongue subsites being the most frequent within oropharynx and oral cavity region, respectively), and more rarely in the nasal cavity and paranasal sinuses, hypopharynx, and larynx (2–4).

The general management of non-nasopharyngeal LEC, so-called LELC (lymphoepithelial-like carcinomas), is a multimodality treatment that includes surgical resection along with neck dissection, radiotherapy, or chemoradiotherapy (5). In resectable LELC of the oropharynx and oral cavity, excision surgery followed by adjuvant radiotherapy or, in selected cases, concurrent adjuvant chemoradiotherapy is the more promising and enacted therapeutic protocol (6).

Abrikossoff’s tumor is a rare mesenchymal neoplasm of neural histogenesis, also known as granular cell tumor (GCT), typically arising in the head and neck region, more commonly in oral cavity mucosa, but virtually ubiquitous both in the cervicofacial region and elsewhere in the trunk, extremities, and, less commonly, in visceral locations. It may display either a benign or a malignant behaviour, showing different specific histological features; thus, surgical resection is mandatory, especially in the case of neoplasms at high risk of malignant transformation (7).

The aim of this case report and systematic literature review is to present a rare case of an HPV/EBV-negative LEC of the palatine tonsil, with metachronous squamous dysplasia and GCT of vocal cords. This case highlights the unique nature of these uncommon concurrent entities and the rarity of their concurrency itself in the head and neck region. The real challenge of this clinical case is the setting and engagement of a proper treatment schedule for different neoplasms in the same patient simultaneously, with the unavoidable need for unique, combined, and organic patient management.

## Case report

A 62-year-old Caucasian female patient, A.R., smoker (48 pack/year), presented at the ENT/HNS department of Policlinico Umberto I—Sapienza University of Rome with a non-painful right lateral cervical swelling, hoarseness, and discomfort while swallowing. The maxillofacial and neck computed tomography (CT) with and without contrast revealed a gross nodular formation (*D*_max_ 4.33mm) at the right lateral cervical level, compatible with nonspecific lymphadenopathy.

A second CT, performed at a different hospital within the next 45 days, revealed, in the right lateral cervical region, a gross solid formation with regular margins and an oval morphology (5 × 4 × 2.5 cm) that shows inhomogeneous counterstain impregnation and the presence of solid tissue at the ipsilateral glossoepiglottic fold (2 × 1 cm). A slightly hypodense thickening is also viable in the left glossoepiglottic fold (*D*_max_ 1 cm). Subcentimetric rounded lymph node formations are observed bilaterally at all remaining neck lymph node stations, which are greater in number and size on the right side.

**Figure 1:**
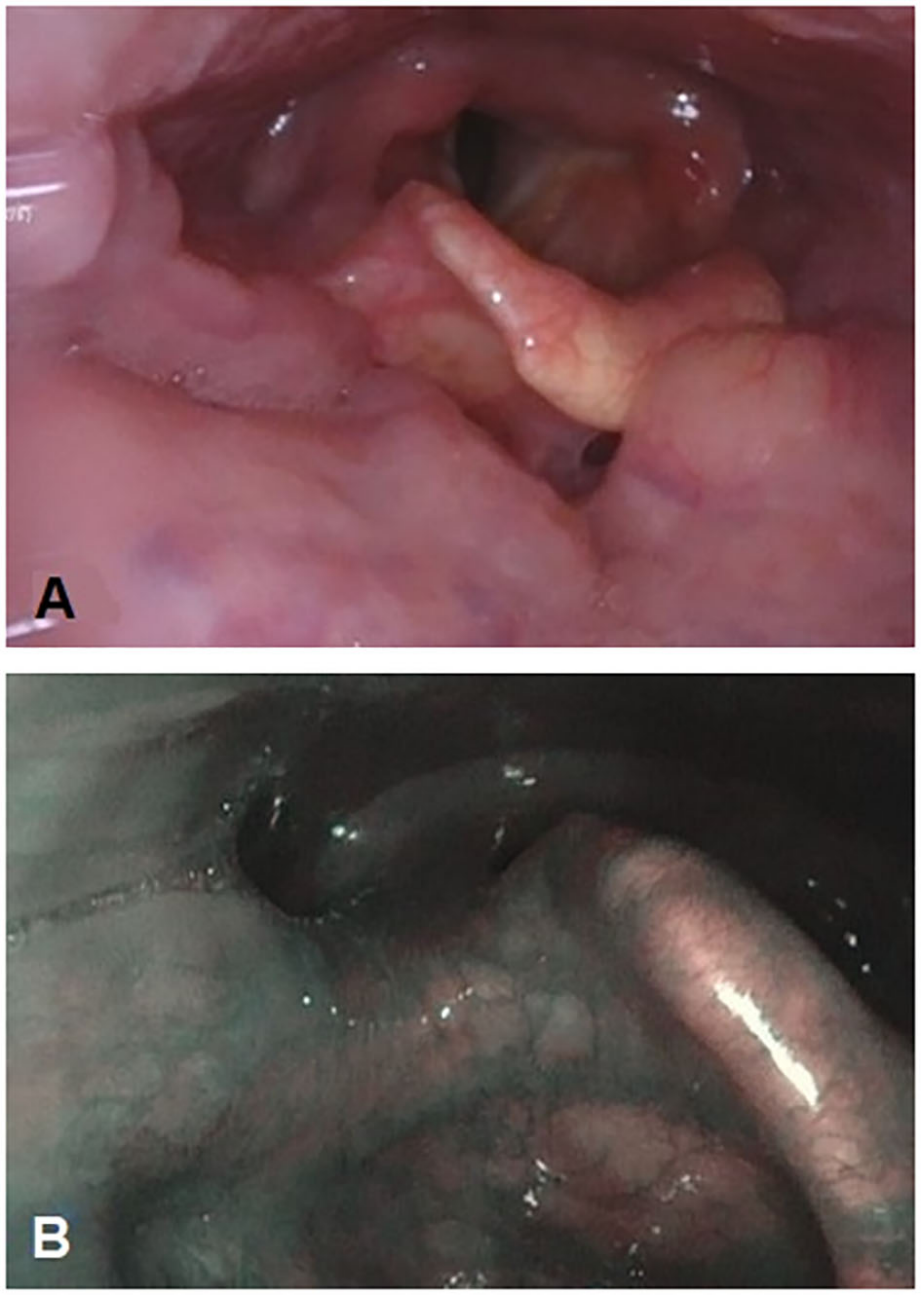
Primitive neoplasm at the level of the right tonsil pillar is endoscopically barely visible with fiber-optic flexible instrument **(A)**. Narrow-band imaging technology integration in endoscopy to better characterize the mucosal aspect and the vascularization of the right tonsil pillar **(B)**.

**Figure 2:**
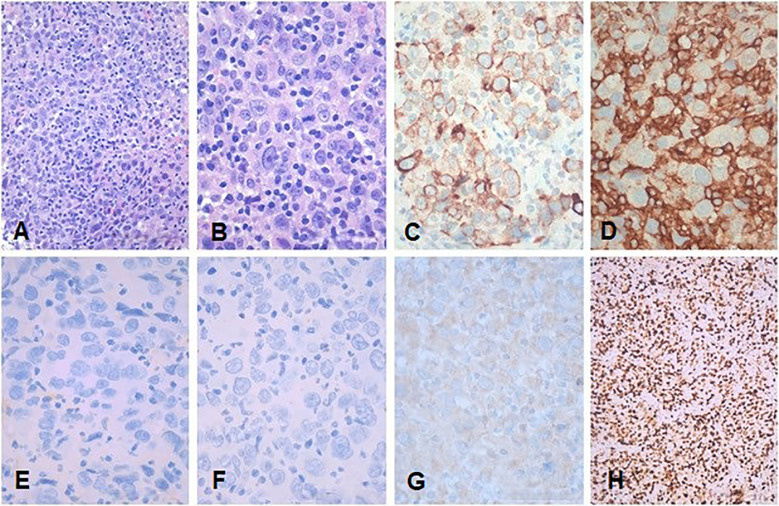
Representative low- and high-power magnification histological images of the tonsillar LEC are shown in **(A, B)**, respectively. **(C)** CKAE1AE3. **(D)** CD45. **(E)** S100. **(F)** EBV (LMP1). **(G)** p16. **(H)** Ki67. Neoplastic cells show immunoreactivity for CKAE1AE3 but not for CD45, S100, and EBV (LMP1). Immunohistochemistry for p16 showed a faint cytoplasmic stain in the absence of any nuclear staining consistent with a negative result according to the definition of AJCC 8th edition (8). **(A, B)** Hematoxylin and eosin. Bars: 100 microns in **(A, H)** and 200 microns in **(B–G)**.

Consequently, a right-palatine-tonsil incisional biopsy was performed, in optic-fiber laryngoscopy vision (**Figure 1A**), with the help of integrated narrow-band imaging (NBI) technology (**Figure 1B**). The histological examination of the harvested tissue revealed a poorly differentiated malignant tumor composed of pleomorphic cells characterized by vesicular nuclei and prominent eosinophilic nucleoli associated with a dense lymphoplasmacytic cell infiltrate (**Figures 2A, B**). Necrosis was focally observed. Neoplastic cells showed immunoreactivity for CK AE1AE3 (**Figure 2C**) but not for CD45 (**Figure 2D**), S100 (**Figure 2E**), synaptophysin, chromogranin, and EBV (LMP1, **Figure 2F**).

Immunohistochemistry for p16 (**Figure 2G**) revealed a faint cytoplasmic staining of the neoplastic cells, but it was considered negative according to the definition of the 8th edition of AJCC (8). Immunostaining for CD45 highlighted the abundant intra-tumoral inflammatory lymphoid component. The proliferative activity (Ki67, **Figure 2H**) was greater than 80%. The pathological findings were consistent with LEC.

**Figure 3:**
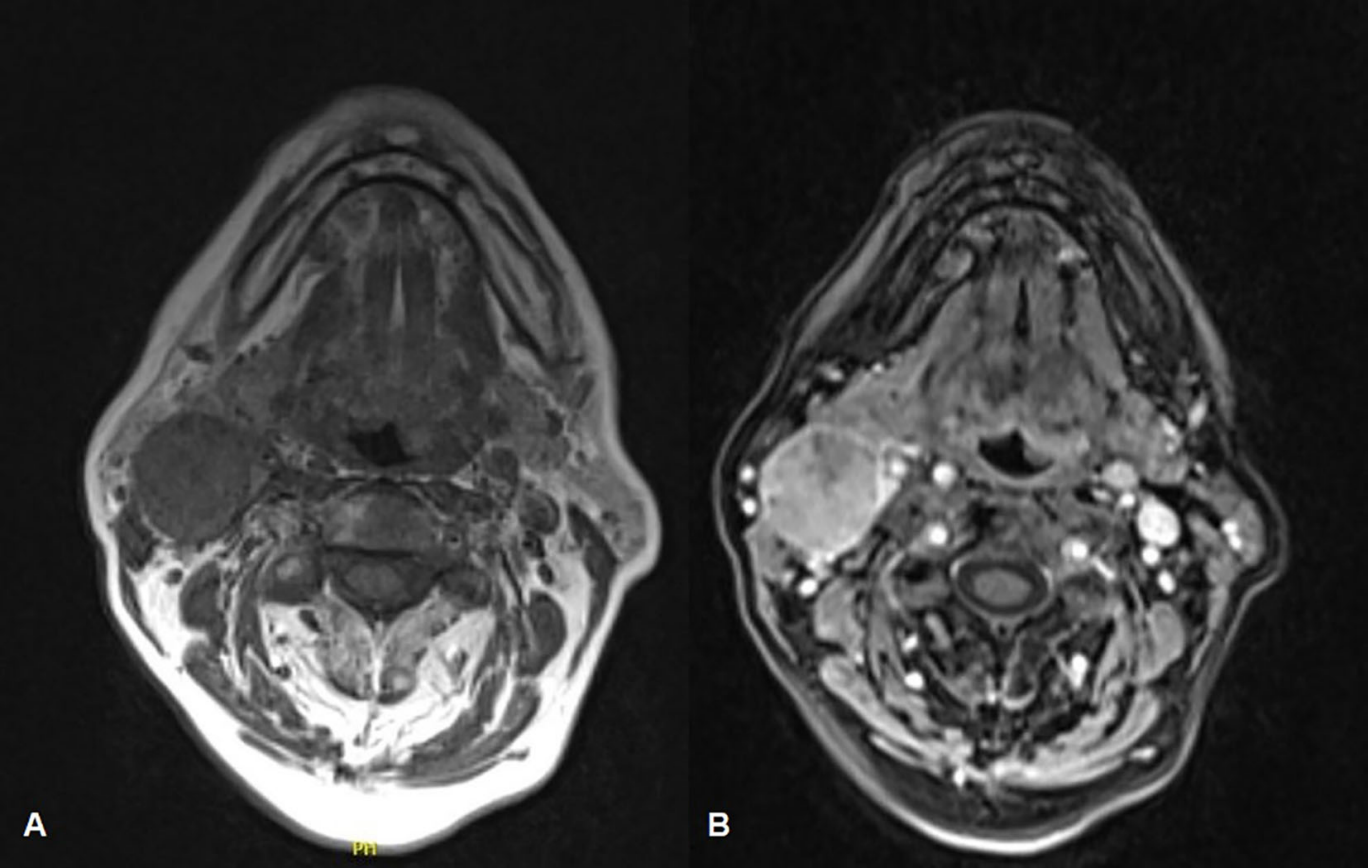
Face-bone MRI showing the primitive neoplasm at the right tonsillar pillar (1 cm), along with numerous lymphadenopathies located bilaterally in the lateral cervical region, within which necrotic-colliquative phenomena are evident **(A)**. A modest post-contrast impregnation is evident at the right tonsillar pillar and the most prominent lymphadenopathy is noted at the right cervical area, to the level IIa **(B)**.

The maxillofacial and neck MRI showed a modest post-contrast impregnation and narrowing in DWI sequences at the right tonsillar pillar (1 cm). Numerous lymphadenopathies are noted bilaterally, the most prominent being in the right lateral cervical level area (IIa level) of approximately 50 × 26 × 25mm, with narrowing in DWI sequences as per ENE+. Necrotic-colliquative phenomena were present within lymphadenopathies (**Figures 3A, B**). Thus, the clinical staging was cT1N2b (9).

The instrumental and histological conclusion was suggestive of tonsillar LEC cT1N2b [according to TNM, AJCC 8th edition guidelines (9)], G3, HPV (p16) negative, and EBV (LMP1) negative.

**Figure 4:**
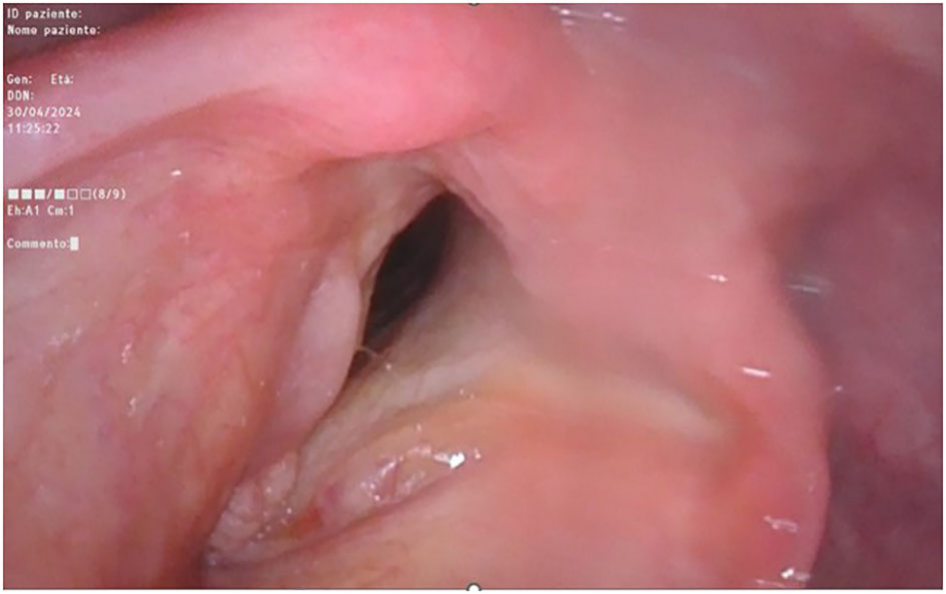
Fiber-optic endoscopic laryngoscopy showing the glottic plane, where is clearly visible the right-true-vocal-cord hypertrophic dysplastic neoplasm and the left-true-vocal-cord granular cell tumor, both at the anterior third level.

On optic-fiber laryngoscopy, while performing excisional tonsillar biopsy, a polypoid neoformation involving the anterior commissure and the anterior third of both true vocal cords was observed (**Figure 4**) by descending endoscopically to the glottic plane from the oropharynx.

**Figure 5:**
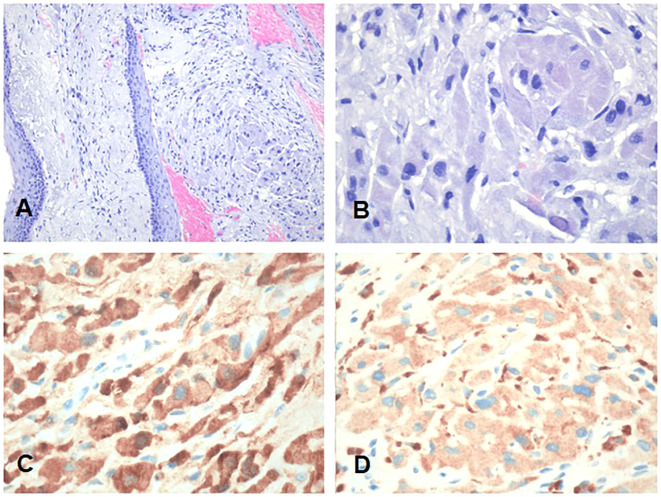
Representative low- and high-power magnification histological images of the granular cell tumor involving the anterior commissure and the anterior third of both true vocal cords are shown in **(A, B)**, respectively. **(C)** S100. **(D)** CD68. Neoplastic cells show a large granular cytoplasm and are immunoreactive for S100 and CD68. **(A, B)** Hematoxylin and eosin. Bars: 100 microns in A and 200 microns in **(B–D)**.

**Figure 6:**
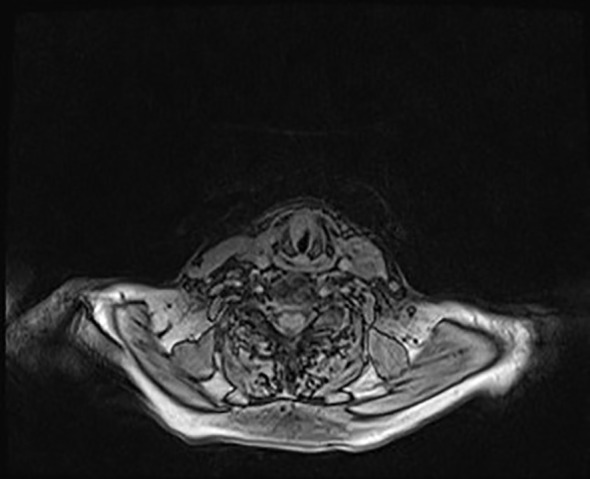
Glottic plane MRI showing the dysplastic hypertrophic neoformation clearly seen at the anterior third of the right true vocal cord while, contralaterally, at the anterior third of the left vocal cord, the neoplasm compatible with granular cell tumor is present, though less viewable.

Thus, the patient underwent surgical excision of the polypoid neoformation in micro-laryngoscopy (MLS) of the polypoid neoformation. At histology, the lesion consisted of large cells with granular cytoplasm (**Figures 5A, B**) that showed immunoreactivity for S100 (**Figure 5C**) and CD68 (**Figure 5D**). These features were consistent with GCT (Abrikossoff’s tumor), as evident in MRI scans of the glottic plane, as shown in **Figure 6**.

The Collegiate Multidisciplinary Head and Neck Tumor Board decided to subject the patient to TORS (transoral robotic surgery) right oropharyngectomy and contralateral tonsillectomy, right radical laterocervical lymph-node emptying, and temporary tracheostomy, followed by adjuvant chemoradiotherapy or radiotherapy. Regarding the glottic left-true vocal cord dysplasia and right-true vocal cord GCT, the Board-shared indication is for surveillance and follow-up since a closed-margin excision in MLS surgery was performed.

The patient underwent surgical intervention with the da Vinci Surgical System, which was performed starting with the execution of a sub-isthmic tracheostomy and open modified radical right lateral cervical lymph node emptying (levels: Ib-II-III-IV-V). A TORS lateral right oropharyngectomy (type II) and contralateral left tonsillectomy was performed.

All the excised tissues were sent for pathologic evaluation, which confirmed the diagnosis of LEC and revealed multiple nodal metastasis (ENE+). Neoplastic vascular invasion and perineural infiltration and ink on tumor were not observed. The final pathologic staging was pT1N3b (9).

The collegial reassessment of the case referred the patient, considering the histological examination and pre-operative imaging, to adjuvant chemoradiotherapy, with prior maxillofacial and neck MRI and HRCT, with and without contrast.

On postoperative follow-up, the patient’s local conditions were good. CT scan of the chest (HRCT) performed 1 month after surgery showed a dimensional increase of some pulmonary nodules, while control MRI of the facial bone and neck, contextually executed, revealed no signs of contrast enhancement of pathological significance at the sites adjacent to the excised tumor, confirming the negativity of the resection margins (R0) and the absence of residual disease in T and the remaining lymph node stations (N). A close follow-up program, with surgical reevaluations and imaging examinations, was planned for the patient, as determined by the Collegial Board.

Clinical monitoring was performed in the following weeks with serial fiberoptic video-endoscopy reevaluations of the upper aerodigestive tract. Six months after the last radiologic follow-up, the reevaluation MRI showed no evidence of pathologic recurrence of disease or post-contrast impregnation in the areas affected by the excised primary tonsillar tumor and showed good surgical outcomes on the laryngeal neoformation.

## Discussion

The LEC of the head and neck region is typically a nasopharyngeal neoplasm, as its histological appearance is analogous to an undifferentiated NPC, as previously asserted. Other subsites of localization of this tumor were rarely described in the few case series reported in the literature at present, which report primary occurrences of LECs other than the nasopharynx, within the head and neck region, identifying these entities as LELCs. Dubey et al. and Ma et al. suggest in their studies that the most frequent localization—other than the nasopharynx—is the oropharynx and salivary glands; fewer localizations to the oral cavity and nasal cavity/paranasal sinuses have also been described (palatine tonsils and tongue subsites are most frequent within the oropharynx and oral cavity regions, respectively) (2, 3).

Epidemiologically, head and neck LEC has a clear male-sex preponderance; Bai et al., in a SEER-based population cohort study (from 1988 and 2013), state that the male-to-female ratio in incidence is 1.6:1, with the white race being more affected than the black population. Advanced disease stages, specifically stages III and IV, are the most common and frequent presentation of LEC among patients, along with lymph-node metastases, which are a likewise common clinical evidence at first diagnosis (10). The median age of incidence is 40 years, and the trend in mortality is worse in elderly patients, a factor that was independently associated with more advanced disease stages and worse survival (11).

The clinical features of these pathological entities are protean and depend on the anatomical subsite of origin of the neoplasm. Symptomatology is generally mild and nonspecific, leading to a delay in diagnosis, especially in NPC cases. As a result, patients with NPC and LELC are often diagnosed at an advanced or metastatic stage of disease. The incidence of lymph node metastasis in LELC is high due to the intrinsic nature of the neoplasm and the diagnostic delay itself (12).

In 2015, Chan et al. reported a cohort of 378 patients in the US with non-NPC head and neck LELC from the SEER database, from 1973 to 2011. The major part were male, white, aged <60 years, with locally advanced/metastatic (stage III–IV) and positive lymph-node disease. The 5-year overall survival (5-y OS) observed in the overall population was 70.5%, while the 5-year disease-specific survival (5-y DSS) was 77.7%. The majority of patients had an oropharyngeal primary LELC, and showed similar OS and DSS to median value (72.1% and 77.4%, respectively), worse than salivary glands primary (5-y OS 80.8%, 5-y DSS 85.7%) and with a slightly better 5-y OS than oral cavity LELC (72.1% vs. 68.8%) (13).

Wang et al. reported a cohort of 179 salivary gland LELC patients in the US from the same database, demonstrating a significantly associated survival with patient and tumor characteristics, which has been shown to be substantially favorable, with a median OS of 206 months (14).

On histological finding of LEC, immunohistochemical testing of cytologic markers for human papillomavirus (HPV) and Epstein–Barr virus (EBV), is a proper in-depth diagnostic investigation, since the tumor may demonstrate, as do squamous cell carcinomas of certain head and neck subsites, an etiopathogenetic link to EBV and HPV infection. While this etiopathogenetic link with viral HPV and EBV infections is clear for HNSCC, in LEC, the presence of EBV is specifically and quite commonly highlighted in endemic areas, while HPV may play a role in upper-digestive-tract primary localizations (5).

The mainstay of the management of head and neck LEC is radiotherapy of locoregional lesions since LECs of the nasopharynx have shown high radiosensitivity, and radiation therapy demonstrated high rates of locoregional control (15). Radiotherapy can likewise be employed simultaneously on lateral cervical lymph nodes if involved. Chemotherapy is typically associated with radiotherapy in more advanced settings of pathology (16); concurrent chemoradiotherapy improves survival in advanced and metastatic NPC (17), while surgery is generally performed as a salvage procedure in recurrent-metastatic setting, either in the primary site or in regional locations of metastasis, having even been shown to be a better alternative in terms of morbidity and quality of life than reirradiation (18), remarkably in patients with no cervical lymph-node metastasis (19).

Surgery plays a crucial role in recurrent-metastatic LEC, and in this context, the use of radiotherapy, whether as an induction or adjuvant approach, is challenging for consolidating the whole therapeutic outcome (13).

GCTs (so-called Abrikossoff’s tumors) are typically asymptomatic and painless neoplasms, generally evident as small-sized (*D*_max_ less than 3 cm), brown-red or skin-colored, slow-growing and solitary nodules (20). Currently, the most likely and accredited histogenesis is from Schwann cells, supported by immunohistochemical analyses and electron microscopy, whereas, in the past, GCT was believed to be of myoblastic origin (21).

The large concentration of peripheral nerves in those anatomic areas of GCT’s origin seems to parallel its neural histogenesis; however, there is evidence of GCTs of non-neural origin, which do not exhibit the S100 pathognomonic marker (22). Laryngeal and vocal fold involvement has been described in less than 10% of the case history in literature (23), presenting as pale masses arising from the submucosal layer at the glottic level or subglottis, with clinical evidence of hoarseness, dysphagia, dyshponia, and globus pharyngeus (24).

Its development is more common in the female sex (with a female-to-male ratio of more than 1.8:1) and in patients younger than 60 years, typically ranging from the fourth to the sixth decade of life (7). The benign and malignant features of GCT are histologically fairly different; macroscopic evidence of necrotic, ulcerated, and hemorrhagic areas, correlated to rapid growth (along with high mitotic index) and a pleomorphic cytological aspect, are usually associated with malignancies, although benign forms are by far the most frequently encountered, relegating malignant masses to less than 1% of overall cases (25).

Fanburg et al. proposed six criteria to determine whether a GC tumor is malignant or benign: the presence of necrosis, the emergence of spindle cells, a vacuolar nucleus with an enlarged nuclear body, an increase in nuclear division, an increase in the nucleoplasmic ratio, and an increase in polymorphisms, suggesting a malignant histological presentation of Abrikossoff’s tumor. If less than three of the previous criteria are met, the tumor is defined as atypical (26).

In a nutshell, a tumor of 3 cm or less in size, which is free of ulcerations, necrotic areas, and local hemorrhagic foci, can be regarded as benign with sufficient certainty (27). Benign forms are by far the most frequently encountered, relegating malignant masses to less than 1% of overall cases (25).

The management of GCT, either benign or malignant, for all anatomic localizations, requires surgical treatment with close margins, and the indication is linked not only to the malignant nature of neoformation, but also to certain issues related to the mass effect (20, 28). The possibility of employment of chemoradiotherapy or immunotherapy in any setting is currently, in light of the limited empirical evidence, controversial (29). Lymph-node dissection is likewise debated; in benign GCT, this surgical approach is widely discouraged, as it may result in unnecessary and is definitely burdened with high morbidity. In malignant tumors, lymph node biopsies are recommended before a complete dissection, which can be favorably followed by adjuvant radiotherapy (30).

## Conclusions

Oropharyngeal LEC and laryngeal GCT are rare localizations of rarer pathologic entities. Their concurrent presence is unique in current literature. While LEC is statistically more commonly malignant, GCT is more often benign. In any case, surgical excision is indicated for both tumors and represents the gold standard in the management of both oncologic entities.

When dealing with LEC, a closed-margin surgery is key to therapeutic success, and contextual lateral cervical emptying and adjuvant radio-chemotherapy are likewise crucial; nearly all the available case series regarding the management of LELCs support this statement. Surgery is also central in the treatment of GCTs. In both cases, close follow-up is mandatory.

Further empirical developments are needed, as well as the breakthrough of novel specific biomarkers and molecular pathways, which will make a tailored target therapy feasible for each patient, aiming to consolidate and enhance the whole therapeutical outcome.

## Data Availability

The raw data supporting the conclusions of this article will be made available by the authors, without undue reservation.
